# Detection of Ascorbic Acid by Two-Dimensional Conductive Metal-Organic Framework-Based Electrochemical Sensors

**DOI:** 10.3390/molecules29112413

**Published:** 2024-05-21

**Authors:** Shi Wang, Ping Li, Junyi Wang, Jun Gong, Helin Lu, Xiaobo Wang, Quan Wang, Ping Xue

**Affiliations:** School of Pharmacy, Xianning Medical College, Hubei University of Science and Technology, Xianning 437100, China

**Keywords:** two-dimensional conductive metal-organic framework, sensor, electrocatalysis, ascorbic acid

## Abstract

The realization of efficient and accurate detection of biomolecules has become a key scientific issue in the field of life sciences. With the rapid development of nanotechnology, electrochemical sensors constructed from the superior physical and chemical properties of nanomaterials show faster and more accurate detection. Among nanomaterials, two-dimensional conductive MOF (2D cMOF) is considered to be a star material in electrochemical sensors due to its remarkable conductivity, high porosity, and stability. In this paper, a Cu_3_(HHTP)_2_/SPE electrochemical sensor for the detection of ascorbic acid (AA) was constructed by modifying 2D cMOF (Cu_3_(HHTP)_2_) on the surface of the screen-printed electrode (SPE). The sensor exhibited excellent catalytic activity in the detection of AA, with a lower detection limit of 2.4 μmol/L (S/N = 3) and a wide linear range of 25–1645 μmol/L. This high catalytic activity can be attributed to the abundant catalytic sites in Cu_3_(HHTP)_2_ and the rapid electron transfer between Cu^+^ and Cu^2+^, which accelerates the oxidation of AA. This work lays a foundation for the subsequent development of MOFs with special electrochemical catalytic properties and the integration of 2D cMOF into intelligent electrical analysis devices.

## 1. Introduction

Ascorbic acid (AA), also known as vitamin C, is an antioxidant that effectively scavenges free radicals in the body to reduce the incidence of cancer, and also plays a crucial role in biological metabolism [1,2,3,4]. It is worth mentioning that both low- and high-AA levels are closely associated with certain diseases. A lack of AA in the body will lead to symptoms such as scurvy and bleeding gums, while excessive intake of AA causes stomach convulsion, diarrhea, and urinary stones [5,6]. Therefore, the development of a simple, low-cost, and high-sensitivity AA detection pathway is essential for the early prevention of these diseases. Among the many detection methods of AA, electrochemical analysis has shown great application prospects due to its high sensitivity and fast response [7,8]. However, it is difficult to determine AA using traditional electrodes such as glassy carbon and platinum as the redox reaction of AA on bare electrodes is irreversible and requires high overpotential [9]. In addition, its high overpotential will cause electrode fouling, resulting in poor selectivity and reproducibility of AA detection [10]. To break this deadlock, more and more studies have been carried out to modify electrodes with suitable electrochemically active materials, including metal nanoparticles, carbon materials, and polymers, for AA detection [11,12,13,14]. Over the past few decades, the rapid development of nanomaterials has made a major breakthrough in terms of the activity of material-based electrochemical sensors [1,15,16], but their sensitivity and stability still need to be improved. Therefore, the further development of innovative electrode modifiers with high specific surface area, multiple active sites, and ultra-high stability is crucial for the detection of AA with high sensitivity.

Metal-organic frameworks (MOFs), as a novel porous material, have attracted extensive attention in the fields of catalysis [17], separation [18], energy storage [19], and drug delivery [20] due to their high porosity, large surface area, and structural diversity. Recently, MOFs have also been used for electrocatalytic sensing, but their inherent low conductivity severely affects the efficiency of charge transport, so they need to be combined with other conductive materials to improve their electrochemical performance [21,22]. However, composite materials will reduce the specific surface area and stability of the MOF, and the composite interface is difficult to accurately control. Excitingly, the emergence of a two-dimensional conductive MOF (2D cMOF) has reversed this impasse. Two-dimensional cMOF not only inherits the inherent advantages of MOF, but is also endowed with high charge carrier mobility and excellent conductivity because of the unique π-π packing and π-d conjugation in their structure [23,24], which is a more ideal electrode modifier. Cu_3_(HHTP)_2_ is a typical 2D cMOF with high electrical conductivity at room temperature and good water stability [25]. Moreover, both the metal center and the ligand of Cu_3_(HHTP)_2_ are redox sites [19], which is expected to further accelerate the electron transport of AA oxidation. All of these imply that Cu_3_(HHTP)_2_-modified electrodes have great potential to be excellent electrochemical sensor for the detection of AA.

In this work, Cu_3_(HHTP)_2_ with high crystallization and specific surface area was synthesized through a simple solvothermal method. Subsequently, a Cu_3_(HHTP)_2_/SPE electrochemical sensor for the detection of AA was constructed by modifying 2D cMOF on the surface of SPE. As expected, the sensor detects AA with high sensitivity and wide linear range. The high catalytic activity of the Cu_3_(HHTP)_2_/SPE sensor can be ascribed to the abundant active sites and high conductivity of Cu_3_(HHTP)_2_, promoting the electron transfer in terms of AA oxidation and reducing reaction overpotential. This work provides a theoretical basis for the construction of 2D cMOF-based electrochemical sensors, and is expected to achieve rapid detection of AA in practical biological samples.

## 2. Results and Discussion

**Figure 1:**
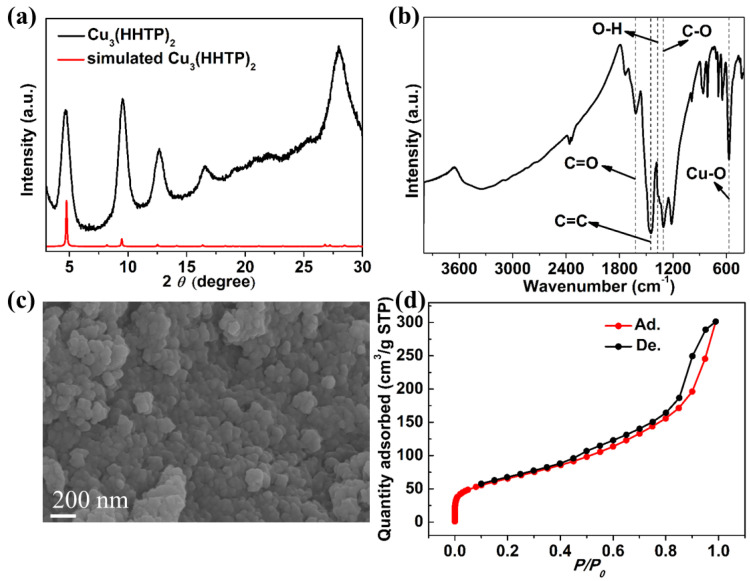
(**a**) The XRD patterns of Cu_3_(HHTP)_2_ and simulated Cu_3_(HHTP)_2_. (**b**) The FT-IR spectrum of Cu_3_(HHTP)_2_. (**c**) The SEM image of Cu_3_(HHTP)_2_. (**d**) The N_2_ adsorption-desorption isotherm of Cu_3_(HHTP)_2_.

The structure and morphology of as-synthesized Cu_3_(HHTP)_2_ was characterized by X-ray diffraction (XRD), Fourier transform infrared (FT-IR) spectrometry and field emission scanning electron microscopy (FE-SEM). As shown in Figure 1a, the XRD pattern of Cu_3_(HHTP)_2_ exhibits three prominent peaks at 4.7°, 9.5°, and 12.8°, corresponding to the (100), (200), and (210) reflection planes. The minor and broad peak at 28° can be attributed to the (004) crystal plane of Cu_3_(HHTP)_2_ [26]. All the peaks of Cu_3_(HHTP)_2_ display good agreement with the reported characterization [27] and simulated data, demonstrating the formation of Cu_3_(HHTP)_2_. In the FT-IR spectrum (Figure 1b), the characteristic bands at 1457 cm^−1^, 1303 cm^−1^, and 575 cm^−1^ belong to the C=C, C-O, and Cu-O vibration in Cu_3_(HHTP)_2_, respectively [28], and the formation of coordination between Cu and O atoms further proves the successful synthesis of Cu_3_(HHTP)_2_. Additionally, the vibration bands located at 1648 cm^−1^ and 1374 cm^−1^ are assigned to the C=O and O-H bonds, where the existence of C=O bonds indicates the partial oxidization of organic ligands (HHTP) in the hydrothermal reaction, while the O-H bonds suggest that a small number of hydroxyl groups are not involved in the coordination reaction [1,19]. The SEM image (Figure 1c) of the obtained Cu_3_(HHTP)_2_ reveals particle-shaped morphology with an average diameter of 40 nm, also matching well with previous reports [26].

It is well known that porous material is conducive to catalytic performance, so a N_2_ adsorption and desorption experiment was performed to explore the pore structure of Cu_3_(HHTP)_2_. As can be seen from Figure 1d, the gas adsorption increases rapidly at low relative pressure, presenting the characteristic Type I isotherm (IUPAC classification); meanwhile, it indicates the microporosity of Cu_3_(HHTP)_2_. In addition, the Brunauer-Emmett-Teller (BET) specific surface area of the obtained Cu_3_(HHTP)_2_ is calculated to be 236 m^2^ g^−1^ and the average pore diameter is about 1.4 nm (Figure S1). The above results confirm the prepared Cu_3_(HHTP)_2_ has a large number of internal porous channels that increase the contact area and efficient electron transport between the catalyst and AA.

**Figure 2:**
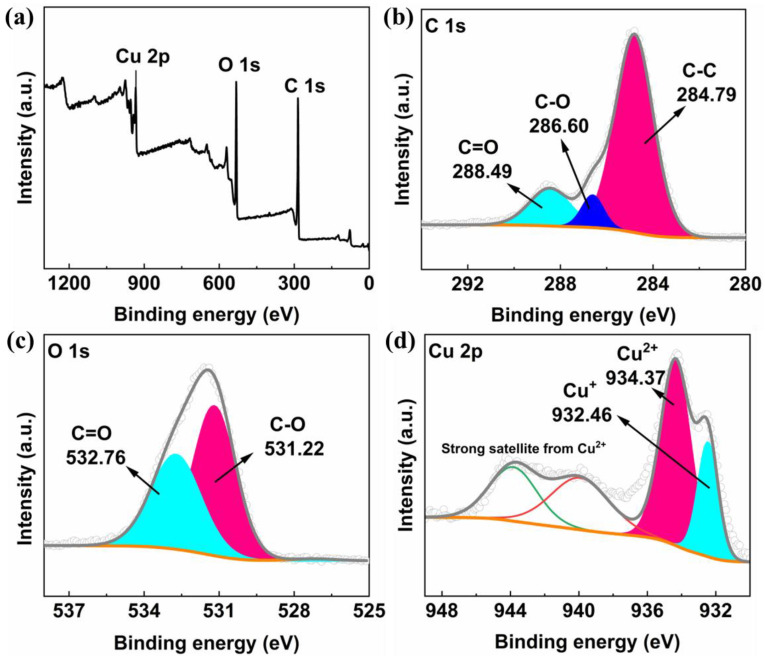
(**a**) The XPS survey spectrum of Cu_3_(HHTP)_2_. (**b**–**d**) The XPS spectra of C 1s, O 1s, and Cu 2p of Cu_3_(HHTP)_2_ (C 1s (**b**); O 1s (**c**); Cu 2p (**d**)).

Next, X-ray photoelectron spectroscopy (XPS) was used to verify the element composition and electronic structure of Cu_3_(HHTP)_2_. The results of the XPS survey spectrum (Figure 2a) reveal the existence of C, O, and Cu elements in the obtained Cu_3_(HHTP)_2_. Moreover, the XPS spectrum of C 1s (Figure 2b) displays three peaks at the binding energy of 288.49, 286.60, and 284.79 eV, which can be attributed to C=O, C-O, and C-C bonds, respectively [29]. As shown in Figure 2c (the XPS spectrum of O 1s), the coexistence of C-O (532.76 eV) and C=O (531.22 eV) [30,31] indicates the formation of semiquinone due to the in situ partial oxidation, as mentioned above (FT-IR results). Furthermore, the peaks at 934.34 and 932.46 eV in the XPS spectrum of Cu 2p (Figure 2d) belong to Cu^2+^ and Cu^+^, respectively [32], and the existence of Cu^+^ indicates unsaturated coordination in Cu_3_(HHTP)_2_ [27,33]. It should be noted that the electronic conductivity of Cu_3_(HHTP)_2_ characterized by the four-point method (Figure S2) is 2.4 S m^−1^. The excellent conductivity of Cu_3_(HHTP)_2_ can be attributed to the fact that the coexistence of Cu^+^ and Cu^2+^ facilitates the delocalization of electrons. The unique structure of 2D cMOF, with high conductivity, is expected to accelerate charge transfer during the electrocatalytic oxidation of AA.

**Figure 3:**
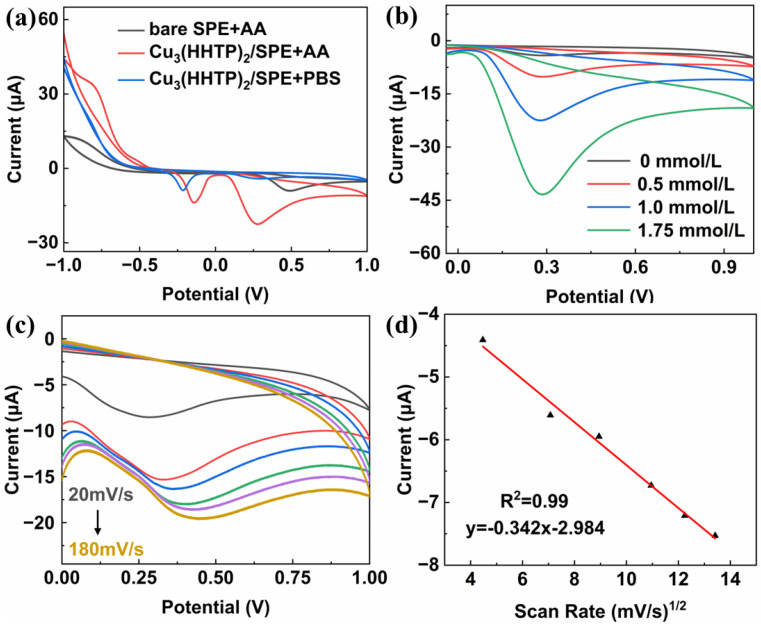
(**a**) The CV curves of bare SPE and Cu_3_(HTTP)_2_/SPE in AA (1 mM) and Cu_3_(HTTP)_2_/SPE in PBS (0.1 mM). (**b**) The CV curves of Cu_3_(HTTP)_2_/SPE in different AA concentrations. (**c**) The CV curves of Cu_3_(HTTP)_2_/SPE at different scan rates. (**d**) The relationship between the current and *v*^1/2^.

To explore the performance of Cu_3_(HHTP)_2_ in the electrocatalytic oxidation of AA, the 2D cMOF was coated on the SPE working electrode to prepare the Cu_3_(HHTP)_2_/SPE sensor, and evaluated by cyclic voltammetry (CV) at the sweep speed of 100 mV/s with or without 1 mM AA. First of all, the condition optimization tests were carried out. The result (Figure S3) exhibits that, within a certain range, the current of the AA oxidation peak increases with the concentration of Cu_3_(HHTP)_2_ (1 to 4 mg/mL). However, with the further increase in concentration, the current of the AA oxidation peak is reversed. The results show that a proper concentration of Cu_3_(HHTP)_2_ effectively enhances the catalytic efficiency of the SPE working electrode, whereas too high concentration will increase the resistivity and affect the performance of the sensor. Furthermore, it can be seen from Figure S4 that the peak current of the AA reaches its maximum at pH = 6 and then gradually decreases with the increase in pH. This can be attributed to the fact that the destruction of the AA intramolecular hydrogen bond under alkaline conditions will reduce its stability and the current of the oxidation peak. Based on this, follow-up electrochemical tests were performed at pH = 6 and a Cu_3_(HHTP)_2_ concentration of 4 mg/mL. As shown in Figure 3a, the CV curve of Cu_3_(HHTP)_2_/SPE exhibits two strong oxidation peaks at 0.28 V and −0.21 V in 0.1 M PBS, which can be assigned to the oxidation potentials of Cu^+^ to Cu^2+^ and C-O to C=O bonds (ligand), respectively, while the peak of bare SPE at 0.48 V is the oxidation of AA. The abundant redox active centers of Cu_3_(HHTP)_2_ and the close oxidation potential between AA and Cu^+^/Cu^2+^ imply Cu_3_(HHTP)_2_ with the ability of accelerating the oxidation of AA. As expected, the oxidation current of AA on Cu_3_(HHTP)_2_/SPE (1.98 × 10^−5^ A) is nearly four times higher than that on bare SPE (5.01 × 10^−6^ A). In addition, in comparison with the initial potential on bare SPE (0.48 V), the lower oxidation potential of AA on Cu_3_(HHTP)_2_/SPE (0.27 V) confirms that the Cu_3_(HHTP)_2_ with outstanding electrocatalytic efficiency promotes the oxidation of AA. Although the above BET results find that Cu_3_(HHTP)_2_ has a high specific surface area, the electroactive surface area (ESA) more directly reflects the effective area of the electrode surface involved in the reaction. Therefore, the ESA of Cu_3_(HHTP)_2_/SPE was tested and calculated using the K_4_[Fe(CN)_6_] system and Randles-Sevcik equation, respectively. The results show that the ESA of Cu_3_(HHTP)_2_/SPE is 0.72 cm^2^ (detailed calculations are provided in the Section 3.2.5), which is significantly larger than that of the traditional inorganic material-modified electrodes [34,35]. This further indicates that Cu_3_(HHTP)_2_-modified SPE possesses more active sites, and as a consequence, it could effectively promote AA oxidation. Based on those and the above structure analysis, the excellent electrochemical performance of Cu_3_(HHTP)_2_ can be ascribed to the following three points: (1) Cu_3_(HHTP)_2_ contains active sites (the coexistence of Cu^+^/Cu^2+^) for AA oxidation; (2) the high conductivity of Cu_3_(HHTP)_2_ accelerates the electron transfer of AA oxidation; (3) different from traditional carbon materials, the unique pore structure of Cu_3_(HHTP)_2_-MOF endows it with a high specific surface area, which provides more active sites for the electrocatalytic oxidation of AA.

To verify the kinetics of the electrocatalytic oxidation, CV tests with different AA concentrations and sweep speeds were carried out. The CV curves of Cu_3_(HHTP)_2_/SPE at different AA concentrations in Figure 3b show that the current increases along with the AA concentration and the oxidation peak moves towards a higher potential with the increase in AA concentration. Moreover, in Figure 3c, with the increase in scan rates, the current gradually increases and the AA oxidation peak shifts in the positive direction. Also, Figure 3d shows that the linear correlation of peak current with *v*^1/2^ can be described as *y* = −0.342*x* − 2.984 (R^2^ = 0.99). These results manifest that the oxidation rate of AA on the Cu_3_(HHTP)_2_/SPE electrode is limited by diffusion-controlled kinetics [36].

**Figure 4:**
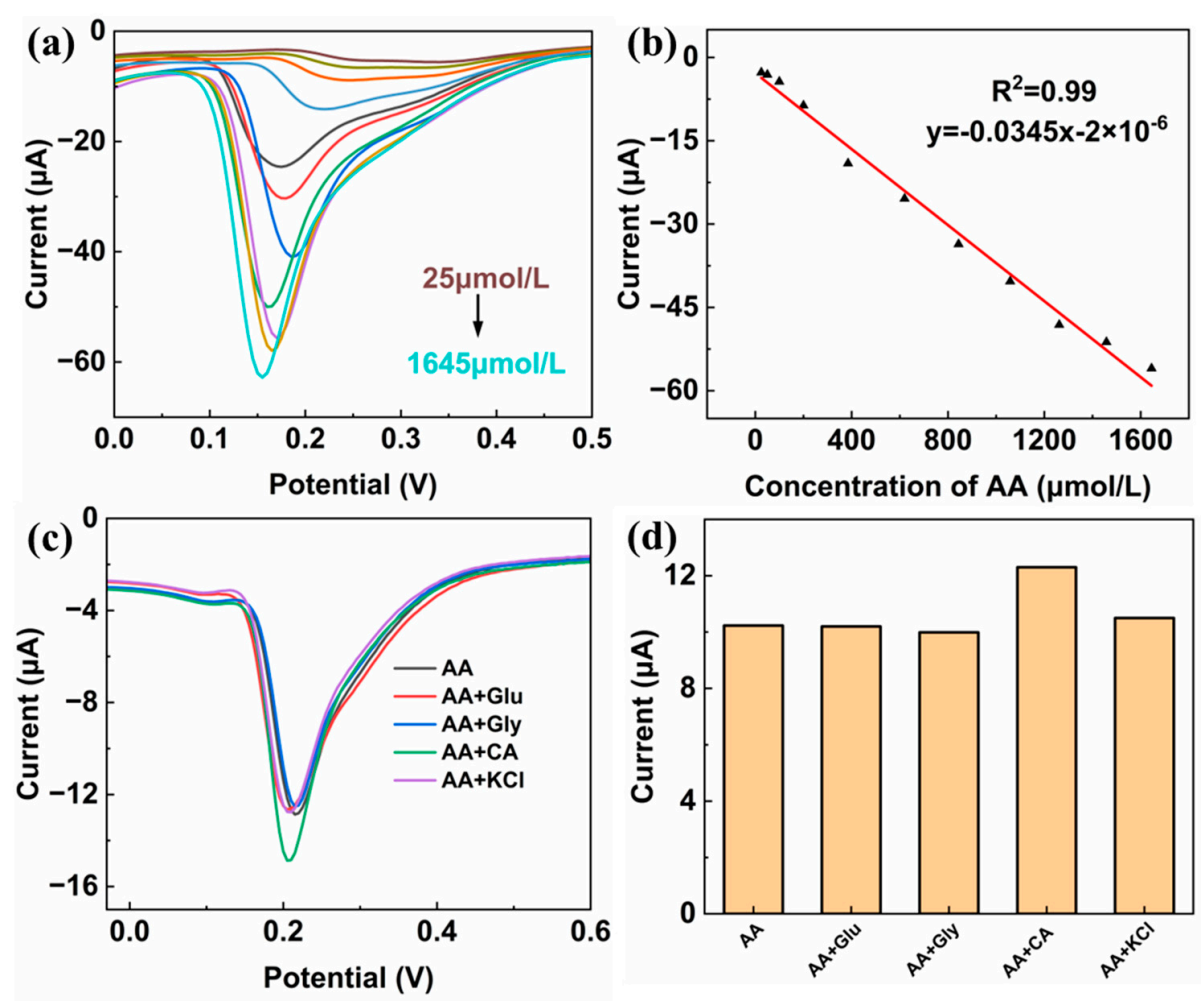
(**a**) The DPV of Cu_3_(HHTP)_2_/SPE for detection of AA with different concentrations. (**b**) The relationship between the current and AA concentration. (**c**) The DPV of Cu_3_(HHTP)_2_/SPE in 200 μM AA with 2 mM KCl, Glu, Gly, and CA, respectively. (**d**) Bar chart of DPV response current of Cu_3_(HHTP)_2_/SPE to AA and AA with interferences.

Then, the detection range and limit of the Cu_3_(HHTP)_2_/SPE sensor were also studied. As exhibited in Figure 4a,b, AA concentration in the range of 25 μmol/L–1645 μmol/L has a certain linear correlation, and the linear equation is as follows: *y* = −0.0345*x* − 2 × 10^−6^ (R^2^ = 0.99). Additionally, the detection limit was 2.44 μmol at a signal-to-noise ratio (S/N) of 3. According to the aforementioned findings, Cu_3_(HHTP)_2_/SPE shows great potential as an excellent AA sensor. Meanwhile, the selectivity and stability of the sensor are also crucial in electrochemical detection, so differential pressure pulse voltammetry (DPV) technologies and current responses at different times are used to evaluate the anti-interference ability and stability of the electrode. In Figure S5a,b, it can be seen that other common interferences (Glu, KCl, Gly, and CA, 10-times interference) also show relatively weak peaks near the oxidation potential of AA. To further explore the selectivity of the sensor to AA, these interferences (10-times interference) were added to AA to verify the effect on current signals of AA. As shown in Figure 4c,d, there is almost no change in the DPV current response of Cu_3_(HHTP)_2_/SPE when AA coexists with other interferences, such as Glu, KCl, and Gly. Although the DPV current signal of AA and CA is 1.2 times higher than that of pure AA, the addition of CA is as high as 2 mM (note that the concentration of CA in the human body is only 0.092 mM [37]), indicating that the interference of CA is negligible. These results show that the sensor possesses high selectivity for AA. Furthermore, the Ip value of Cu_3_(HHTP)_2_/SPE is still as high as 86% (relative to the initial current) after 5 days (Figure S6a,b); as a result, the sensor has high stability. Moreover, the feasibility of the Cu_3_(HHTP)_2_/SPE sensor for the detection of AA in actual serum samples (mouse serum) was verified by standard addition experiments. As shown in Table 1, the relative standard deviations and recovery measured at AA concentrations of 25, 55, and 285 μmol/L are 4.87%/98.6%, 4.08%/98.0%, and 0.67%/99.0%, respectively, indicating that the constructed Cu_3_(HHTP)_2_/SPE sensor can be extended to practical applications and a general adaptative platform can be built for later disease diagnosis. Furthermore, to further verify the stability and reproducibility of the Cu_3_(HHTP)_2_/SPE sensor, electrode uncertainty experiments were also carried out. The results further suggest that the sensor has good stability as a consequence of the low uncertainty of 0.171 (Table S1).

**Scheme 1:**
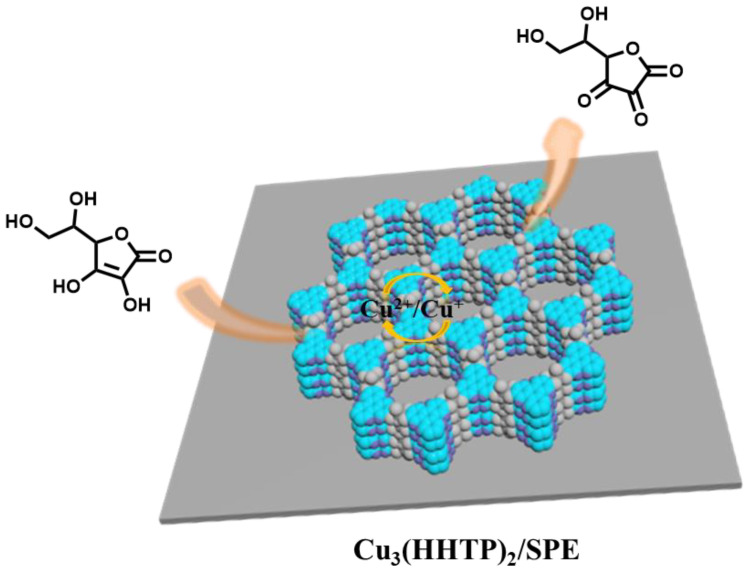
Proposed mechanism of a Cu_3_(HHTP)_2_/SPE sensor for AA detection.

Finally, according to the above XPS and CV results, a possible catalytic mechanism is proposed (Scheme 1). The Cu^+^/Cu^2+^ in Cu_3_(HHTP)_2_ are the activity sites of the electrochemical sensor. During the oxidation of AA, Cu^+^ loses an electron to become Cu^2+^, and then Cu^2+^ gains an electron from AA to promote the oxidation. The electrode material with high conductivity accelerates the electron transfer of the AA oxidation and endows the sensor with a better electrochemical signal.

## 3. Experiment

### 3.1. Instruments and Reagents

The CHI660E electrochemical workstation (Shanghai Chenhua, Shanghai, China), TE100-SG type screen printing electrode (Zensor, Taiwan), FEI Talos F200X Field Emission transmission electron microscope (FEI Corporation, Hillsboro, OR, USA), Thermo escalab 250Xi X-ray Photoelectron Spectrometer (Thermo Fisher Scientific, Waltham, MA, USA), D8 ADVANCE X-ray diffractometer (Bruker, Ettlingen, Germany), IRAffinity-1 Fourier Transform Infrared Spectrometer (Shimadzu, Tokyo, Japan), and the S4800 Field emission scanning electron microscope (Hitachi, Tokyo, Japan) were used in the study. A four-point probe method was used to test electrical conductivity of conductive MOF by SourceMeter (2634B, KEITHLEY, Solon, OH, USA). The nitrogen adsorption-desorption isotherms were measured by an automatic Micromeritics apparatus at 77 K (ASAP2460-Vapor, Micromeritics Instrument Corporation (Norcross, GA, USA); before the test, all samples were degassed under vacuum at 100 °C for 12 h).

The materials were as follows: copper acetate monohydrate (Cu(OAc)_2_-H_2_O), anhydrous ferric chloride (FeCl_3_), ethyl acetate (EA), anhydrous sodium sulfate (Na_2_SO_4_), and o-anisidine dimethyl ether (ODMB) (Shanghai Aladdin Biochemical Science and Technology Co., Ltd., Shanghai, China); dichloromethane (DCM), acetone (C_3_H_6_O), and methanol (CH_4_O) (Shanghai Macklin Biochemical Technology Co., Ltd., Shanghai, China); concentrated sulfuric acid (H_2_SO_4_), boron tribromide (BBr_3_), concentrated hydrochloric acid (HCl), and uric acid (UA) (Sinopharm Chemical Reagent Co., Ltd., Shanghai, China); citric acid (CA), glucose (Glu), and glycine (Gly) (Shanghai Yuanye Biotechnology Co., Ltd., Shanghai, China); ascorbic acid (AA, Tianjin Fuchen Chemical Reagent Factory, Tianjin, China); phosphate buffer solution dry powder (PBS, Beijing Lanjeko Technology Co., Ltd., Beijing, China). All reagents used are analytically pure. All animal handling and experiments were reviewed and approved by the Institutional Animal Care and Use Committee of Hubei University of Science and Technology (approval No. 202303301) in March 2023.

### 3.2. Experimental Methods

#### 3.2.1. Electrode Pretreatment

The screen-printed electrode (SPE) was connected to the electrochemical workstation. Cyclic voltammetric tests were performed in 0.5 mol/L H_2_SO_4_ solution with a scanning voltage range of −1.0~1.0 V and a scanning rate of 100 mV/s until the baseline stabilized, and then the test ended. The electrode surface was rinsed with deionized water and blow-dried.

#### 3.2.2. The Synthesis of Cu_3_(HHTP)_2_

A solvothermal method was used to synthesize Cu_3_(HHTP)_2_. First, 0.320 g of Cu(OAc)_2_-H_2_O and 0.260 g of HHTP were weighed and dissolved in 100 mL and 125 mL of CH_4_O, respectively. Then, the above two solutions were mixed and stirred for 10 min at room temperature, transferred to a sealed volumetric flask, and placed in a 65 °C blast drying oven for 24 h to obtain blue-black solid. The solid was washed by methanol and acetone. Finally, it was dried in a vacuum at 80 °C for 12 h to obtain the Cu_3_(HHTP)_2_.

#### 3.2.3. The Preparation of Cu_3_(HHTP)_2_/SPE

4 mg of Cu_3_(HHTP)_2_ was placed into 1 mL of CH_4_O and ultrasonicated for 30 min for uniform dispersion. Subsequently, 2 μL of the dispersion was applied dropwise on the surface of the working electrode of the SPE. Then, it was dried at room temperature to obtain the Cu_3_(HHTP)_2_/SPE, which was stored at room temperature for spare use.

#### 3.2.4. Electrochemical Test

Cyclic voltammetry (CV) and differential pressure pulse voltammetry (DPV) were carried out using an electrochemical workstation CHI660E with a solution of 0.1 mol/L PBS. The CV had a range of −1 to 1 V and a scanning rate of 100 mV/s. The DPV scanning range was from −1 to 1 V, with a potential increment of 0.005 V, pulse amplitude of 0.05 V, and pulse width of 0.3 s.

#### 3.2.5. Calculation of Electrochemically Surface Area (ESA)

The ESA of Cu_3_(HHTP)_2_/SPE was determined and calculated according to the literature [38]. The ESA of Cu_3_(HHTP)_2_/SPE was estimated using CV. The CV experiments of 5 mM K_4_[Fe(CN)_6_] in 0.1 M KCl solution were performed at working electrode at various scan rates (20, 50, 80, 120, 150, 180 mv/s).

Ip = 2.69 × 10^5^AD^1/2^n^3/2^v^1/2^C

Ip = 2.46 × 10^5^v^1/2^ + 4.66 × 10^−6^ A; D = 0.673 × 10^−5^ cm^2^ s^−1^; n = 1; C_o_ = 5 × 10^−6^ mol·cm^−3^

A = 0.07 cm^2^.

#### 3.2.6. Electrode Uncertainty Experiments

Twelve Cu_3_(HHTP)_2_/SPE sensors were prepared to detect AA. The average current and electrode uncertainty is calculated according to the guideline [39].

$\bar{I}=10.725\mu A$; $U=\sqrt{\frac{\sum_{k=1}^{n}{\left(I_{k}-\bar{I}\right)}^{2}}{n(n-1)}}$

## 4. Conclusions

In this experiment, Cu_3_(HHTP)_2_ was synthesized and then deposited on the surface of SPE to prepare a Cu_3_(HHTP)_2_/SPE sensor for the detection of AA. The sensor’s linear range of AA detection was 25–1645 μmol/L, with the linear equation of *y* = −0.0345*x* − 2 × 10^−6^ (R^2^ = 0.99) and the detection limit of 2.44 μmol/L (S/N = 3). The sensor also showed good anti-interference, reproducibility, and stability. In addition, the sensor was used to detect AA in actual mouse serum samples, which presented good recoveries, confirming the feasibility of the sensor for AA detection in real biological samples.

## Figures and Tables

**Table 1:** Detection of AA in actual serum samples.

Samples	Actual Value (μmol/L)	Detection Value (μmol/L)	RSD (%)	Recovery (%)
Mouse serum	25	24.7	4.87	98.6
	55	53.9	4.08	98.0
	285	282.2	0.67	99.0

## Data Availability

Data are contained within the article and Supplementary Materials.

## Supplementary Materials

The following supporting information can be downloaded at: https://www.mdpi.com/article/10.3390/molecules29112413/s1, Figure S1: Pore size distributions of Cu3(HHTP)2.; Figure S2: Four-point probe I-V curves of Cu_3_(HHTP)_2_.; Figure S3: Effects of Cu_3_(HHTP)_2_ concentration on the electrocatalytic performance of Cu_3_(HHTP)_2_/SPE sensor.; Figure S4: Effects of pH on the electrocatalytic performance of Cu_3_(HHTP)_2_/SPE sensor.; Figure S5: (a) The CV curves of Cu_3_(HTTP)_2_/SPE in AA at different times; (b) Bar chart of AA response current of Cu_3_(HTTP)_2_/SPE at different times.; Table S1: Parallel experiments of Cu3(HHTP)2/SPE sensors.

## References

[1] Liang X.-H., Yu A.-X., Bo X.-J., Du D.-Y., Su Z.-M. (2023). Metal/covalent-organic frameworks-based electrochemical sensors for the detection of ascorbic acid, dopamine and uric acid. Coord. Chem. Rev..

[2] Reang J., Sharma P.C., Thakur V.K., Majeed J. (2021). Understanding the Therapeutic Potential of Ascorbic Acid in the Battle to Overcome Cancer. Biomolecules.

[3] Pizzicannella J., Marconi G.D., Guarnieri S., Fonticoli L., Della Rocca Y., Konstantinidou F., Rajan T.S., Gatta V., Trubiani O., Diomede F. (2021). Role of ascorbic acid in the regulation of epigenetic processes induced by *Porphyromonas gingivalis* in endothelial-committed oral stem cells. Histochem. Cell Biol..

[4] Feng L.-L., Wu Y.-X., Zhang D.-L., Hu X.-X., Zhang J., Wang P., Song Z.-L., Zhang X.-B., Tan W. (2017). Near Infrared Graphene Quantum Dots-Based Two-Photon Nanoprobe for Direct Bioimaging of Endogenous Ascorbic Acid in Living Cells. Anal. Chem..

[5] Guo X., Yue G., Huang J., Liu C., Zeng Q., Wang L. (2018). Label-Free Simultaneous Analysis of Fe(III) and Ascorbic Acid Using Fluorescence Switching of Ultrathin Graphitic Carbon Nitride Nanosheets. ACS Appl. Mater. Interfaces.

[6] Hu G., Ge L., Li Y., Mukhtar M., Shen B., Yang D., Li J. (2020). Carbon dots derived from flax straw for highly sensitive and selective detections of cobalt, chromium, and ascorbic acid. J. Colloid Interface Sci..

[7] Motsaathebe P.C., Fayemi O.E. (2022). Electrochemical Detection of Ascorbic Acid in Oranges at MWCNT-AONP Nanocomposite Fabricated Electrode. Nanomaterials.

[8] Dodevska T., Hadzhiev D., Shterev I. (2023). A Review on Electrochemical Microsensors for Ascorbic Acid Detection: Clinical, Pharmaceutical, and Food Safety Applications. Micromachines.

[9] Argoubi W., Rabti A., Ben Aoun S., Raouafi N. (2019). Sensitive detection of ascorbic acid using screen-printed electrodes modified by electroactive melanin-like nanoparticles. RSC Adv..

[10] Dong Y.P., Huang L., Zhang J., Chu X.F., Zhang Q.F. (2012). Electro-oxidation of ascorbic acid at bismuth sulfide nanorod modified glassy carbon electrode. Electrochim. Acta.

[11] Hatamie A., Rahmati R., Rezvani E., Angizi S., Simchi A. (2019). Yttrium Hexacyanoferrate Microflowers on Freestanding Three-Dimensional Graphene Substrates for Ascorbic Acid Detection. ACS Appl. Nano Mater..

[12] Alvin E.A., de Moraes N.C., de Oliveira Y.M., da Silva D.G.P., Barbosa A.I.D.S., Ribeiro W.S.M., Vermelho M.V.D., de Freitas J.M.D., de Freitas J.D., Dantas N.O. (2024). A novel paper-based electrochemical device modified with Ni-doped TiO_2_ nanocrystals-decorated graphene oxide for ascorbic acid detection. Mater. Chem. Phys..

[13] Jiang C., Xie L., Yan F., Liang Z., Liang J., Huang K., Li H., Wang Y., Luo L., Li T. (2023). A novel electrochemical aptasensor based on polyaniline and gold nanoparticles for ultrasensitive and selective detection of ascorbic acid. Anal. Methods.

[14] Mazzara F., Patella B., Aiello G., O’Riordan A., Torino C., Vilasi A., Inguanta R. (2021). Electrochemical detection of uric acid and ascorbic acid using r-GO/NPs based sensors. Electrochim. Acta.

[15] Fu D., Liu H., Chen T., Cheng Y., Cao M., Liu J. (2022). A bio-analytic nanoplatform based on Au post-functionalized CeFeO_3_ for the simultaneous determination of melatonin and ascorbic acid through photo-assisted electrochemical technology. Biosens. Bioelectron..

[16] Geleta G.S. (2024). Recent advances in electrochemical sensors based on molecularly imprinted polymers and nanomaterials for detection of ascorbic acid, dopamine, and uric acid: A review. Sens. Bio-Sens. Res..

[17] Xue P., Huang J., Lin L., Li R., Tang M., Wang Z. (2021). Zirconium-based metal-organic Framework as an Efficiently Heterogeneous Photocatalyst for Oxidation of Benzyl Halides to Aldehydes. Mol. Catal..

[18] Chen X., Fang S., Xue P., Huang J., Tang M., Wang Z. (2023). Reversible Regulation of Polar Gas Molecules by Azobenzene-Based Photoswitchable Metal-Organic Framework Thin Films. Molecules.

[19] Chen Y., Zhu Q., Fan K., Gu Y., Sun M., Li Z., Zhang C., Wu Y., Wang Q., Xu S. (2021). Successive Storage of Cations and Anions by Ligands of π-d-Conjugated Coordination Polymers Enabling Robust Sodium-Ion Batteries. Angew. Chem. Int. Ed..

[20] Rabiee N. (2023). Sustainable metal-organic frameworks (MOFs) for drug delivery systems. Mater. Today Commun..

[21] Wang Y., Chen J., Wang C., Zhang L., Yang Y., Chen C., Xie Y., Zhao P., Fei J. (2023). An electrochemical sensor based on Ce-MOF-derived Ce-doped poly(3,4-ethylenedioxythiophene) composite for efficient determination of rutin in food. Talanta.

[22] Li Y., Ye W., Cui Y., Li B., Yang Y., Qian G. (2020). A metal-organic frameworks@ carbon nanotubes based electrochemical sensor for highly sensitive and selective determination of ascorbic acid. J. Mol. Struct..

[23] Song X., Wang X., Li Y., Zheng C., Zhang B., Di C.-A., Li F., Jin C., Mi W., Chen L. (2020). 2D Semiconducting Metal-Organic Framework Thin Films for Organic Spin Valves. Angew. Chem. Int. Ed..

[24] Mao P., Lan G., Liu C., Wang Z., Liu Y., Sun H., Huang W. (2021). Recent advances in synthesis of two-dimensional conductive metal-organic frameworks and their electrochemical energy storage application. Sustain. Mater. Technol..

[25] Hmadeh M., Lu Z., Liu Z., Gándara F., Furukawa H., Wan S., Augustyn V., Chang R., Liao L., Zhou F. (2012). New Porous Crystals of Extended Metal-Catecholates. Chem. Mater..

[26] Koo W.-T., Kim S.-J., Jang J.-S., Kim D.-H., Kim I.-D. (2019). Catalytic Metal Nanoparticles Embedded in Conductive Metal-Organic Frameworks for Chemiresistors: Highly Active and Conductive Porous Materials. Adv. Sci..

[27] Xue P., Pan X., Huang J., Gao Y., Guo W., Li J., Tang M., Wang Z. (2021). In Situ Fabrication of Porous MOF/COF Hybrid Photocatalysts for Visible-Light-Driven Hydrogen Evolution. ACS Appl. Mater. Interfaces.

[28] Zhou W., Lv S., Liu X., Li Y., Liu J. (2019). A Directly Grown Pristine Cu-CAT Metal-organic Framework as an Anode Material for High-energy Sodium-ion Capacitors. Chem. Commun..

[29] Zhang Y.-P., Tang H.-L., Dong H., Gao M.-Y., Li C.-C., Sun X.-J., Wei J.-Z., Qu Y., Li Z.-J., Zhang F.-M. (2020). Covalent-organic Framework Based Z-scheme Heterostructured Noble-metal-free Photocatalysts for Visible-light-driven Hydrogen Evolution. J. Mater. Chem. A.

[30] Li C.-C., Gao M.-Y., Sun X.-J., Tang H.-L., Dong H., Zhang F.-M. (2020). Rational Combination of Covalent-organic Framework and Nano TiO_2_ by Covalent Bonds to Realize Dramatically Enhanced Photocatalytic Activity. Appl. Catal. B Environ..

[31] Yao Y.-H., Li J., Zhang H., Tang H.-L., Fang L., Niu G.-D., Sun X.-J., Zhang F.-M. (2020). Facile Synthesis of a Covalently Connected rGO-COF Hybrid Material by in situ Reaction for Enhanced Visible-light Induced Photocatalytic H_2_ Evolution. J. Mater. Chem. A.

[32] Zhang Y.C., Tang J.Y., Wang G.L., Zhang M., Hu X.Y. (2006). Facile synthesis of submicron Cu_2_O and CuO crystallites from a solid metallorganic molecular precursor. J. Cryst. Growth.

[33] Liu Y., Liu M., Shang S., Gao W., Wang X., Hong J., Hua C., You Z., Liu Y., Chen J. (2023). Recrystallization of 2D C-MOF Films for High-Performance Electrochemical Sensors. ACS Appl. Mater. Interfaces.

[34] Uzak D., Atiroğlu A., Atiroğlu V., Çakıroğlu B., Özacar M. (2020). Reduced Graphene Oxide/Pt Nanoparticles/Zn-MOF-74 Nanomaterial for a Glucose Biosensor Construction. Electroanalysis.

[35] Singh S., Meena V.K., Mizaikoff B., Singh S.P., Suri C.R. (2016). Electrochemical sensing of nitro-aromatic explosive compounds using silver nanoparticles modified electrochips. Anal. Methods.

[36] Wang L., Pan L., Han X., Ha M.N., Li K., Yu H., Zhang Q., Li Y., Hou C., Wang H. (2022). A portable ascorbic acid in sweat analysis system based on highly crystalline conductive nickel-based metal-organic framework (Ni-MOF). J. Colloid Interface Sci..

[37] Yoshimi N., Futamura T., Kakumoto K., Salehi A.M., Sellgren C.M., Holmén-Larsson J., Jakobsson J., Pålsson E., Landén M., Hashimoto K. (2016). Blood metabolomics analysis identifies abnormalities in the citric acid cycle, urea cycle, and amino acid metabolism in bipolar disorder. BBA Clin..

[38] Cui H.-F., Ye J.-S., Zhang W.-D., Li C.-M., Luong J.H.T., Sheu F.-S. (2007). Selective and sensitive electrochemical detection of glucose in neutral solution using platinum-lead alloy nanoparticle/carbon nanotube nanocomposites. Anal. Chim. Acta.

[39] EDQM Genera European OMCL Network Quality Management Document: PA/PH/OMCL (18) 145 R1 CORR Evaluation of Measurement Uncertainty (Core Document) [S]. European: Guideline, 2020: 1–9. https://www.edqm.eu/documents/52006/128968/omcl-evaluation-of-measurement-uncertainty-core-document.pdf/b9b88e44-e831-4221-9926-d8c6e8afe796?t=1628491784923.

