# Supplementary material for: Detection of Ascorbic Acid by Two-Dimensional Conductive Metal-Organic Framework-Based Electrochemical Sensors
Source: Molecules. 2024 May 21;29(11):2413. doi: 10.3390/molecules29112413 (PMC11173493; doi:10.3390/molecules29112413)
Supplement: Supplementary file 1 [file molecules-29-02413-s001.zip › molecules-2995517-supplementary.pdf]

molecules

MDPI

Supplementary Materials

Article

Detection of Ascorbic Acid by Two-Dimensional Conductive Metal–Organic Framework-Based Electrochemical Sensors

Shi Wang †, Ping Li †, Junyi Wang, Jun Gong, Helin Lu, Xiaobo Wang, Quan Wang * and Ping Xue *

School of Pharmacy, Xianning Medical College, Hubei University of Science and Technology, Xianning 437100, China; hkwangshi@hbust.edu.cn (S.W.); liping232022@outlook.com (P.L.); waonihaoa1@outlook.com (J.W.); gongjun@hbust.edu.cn (J.G.); hllu@hbust.edu.cn (H.L.); eas7on@163.com (X.W.)
* Correspondence: wangquan@hbust.edu.cn (Q.W.); pingxue@hbust.edu.cn (P.X.)
† These authors contributed equally to this work.

Citation: Wang, S.; Li, P.; Wang, J.; Gong, J.; Lu, H.; Wang, X.; Wang, Q.; Xue, P. Detection of Ascorbic Acid by Two-Dimensional Conductive Metal–Organic Framework-Based Electrochemical Sensors. *Molecules* **2024**, *29*, 2413. https://doi.org/10.3390/molecules29112413

Academic Editor: Dong-Kyun Seo

Received: 18 April 2024
Revised: 9 May 2024
Accepted: 16 May 2024
Published: 21 May 2024

Copyright: © 2024 by the authors. Submitted for possible open access publication under the terms and conditions of the Creative Commons Attribution (CC BY) license (https://creativecommons.org/licenses/by/4.0/).

Table S1. Parallel experiments of Cu3(HHTP)2/SPE sensors.

	Current of oxidation peak (μA)		Current of oxidation peak (μA)
1	9.44	7	11.57
2	10.80	8	10.32
3	10.99	9	11.65
4	11.10	10	10.50
5	10.78	11	10.28
6	10.70	12	10.58

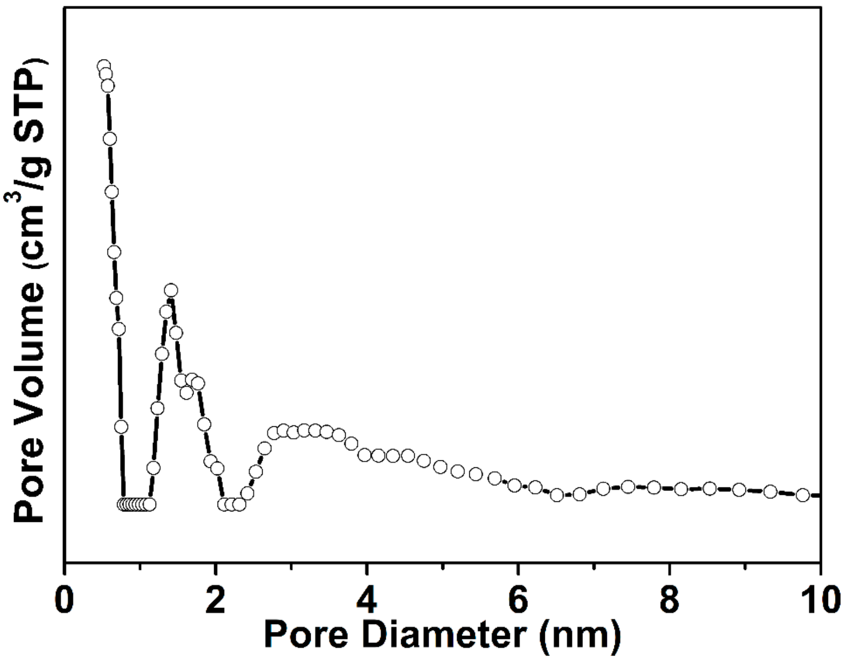

Figure S1. Pore size distributions of $Cu_3(HHTP)_2$.

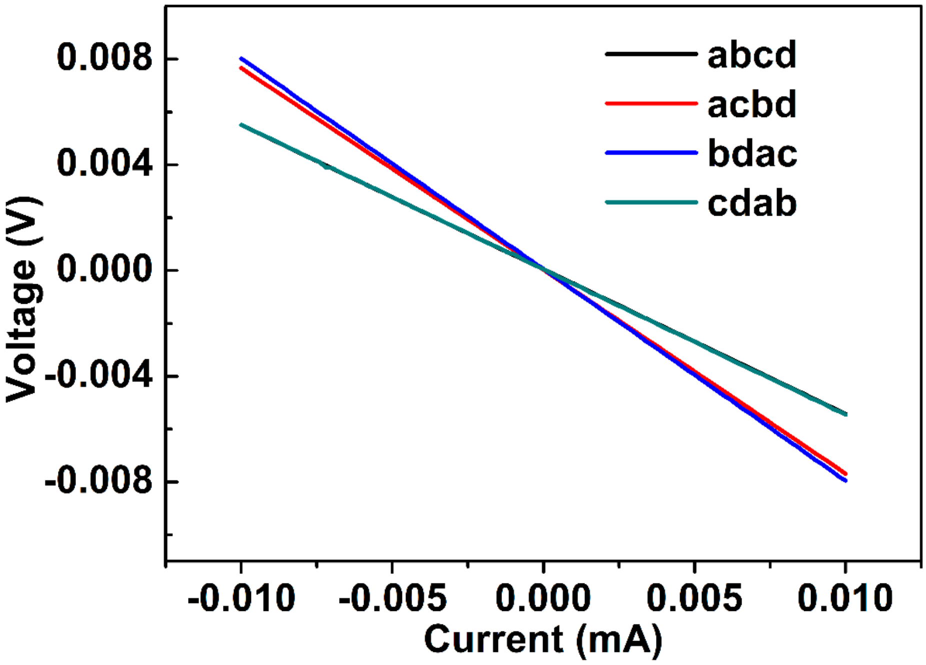

Figure S2. Four-point probe I-V curves of $Cu_3(HHTP)_2$.

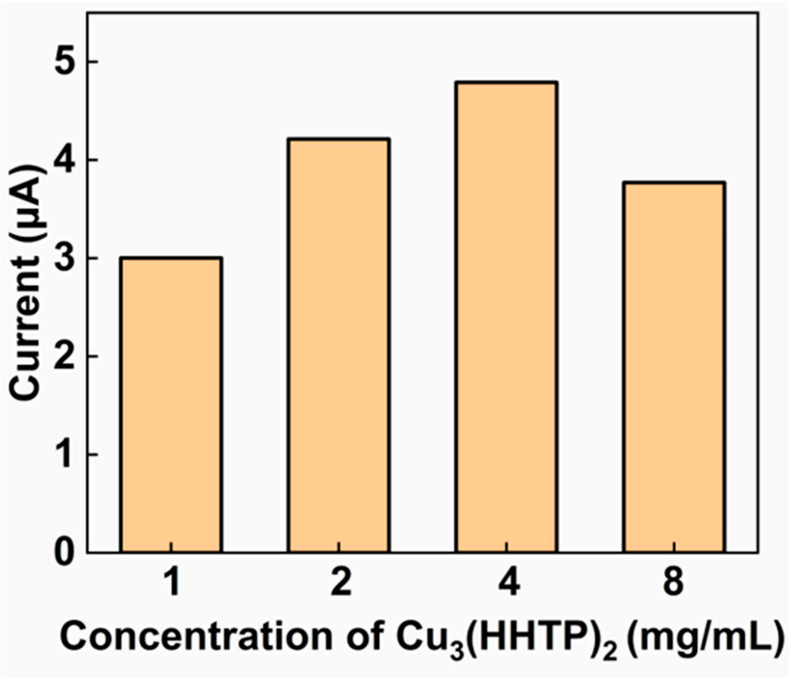

Figure S3. Effects of $Cu_3(HHTP)_2$ concentration on the electrocatalytic performance of $Cu_3(HHTP)_2$/SPE sensor.

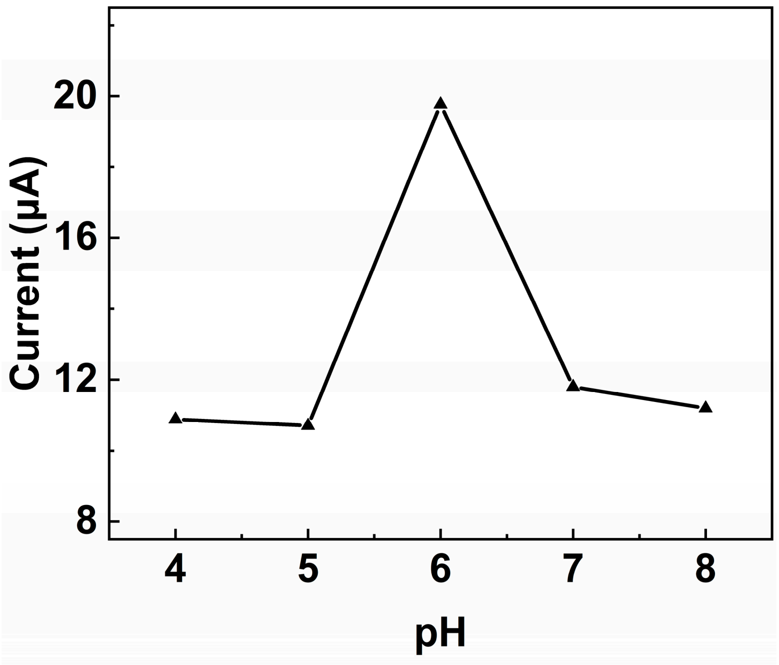

Figure S4. Effects of pH on the electrocatalytic performance of $Cu_3(HHTP)_2$/SPE sensor.

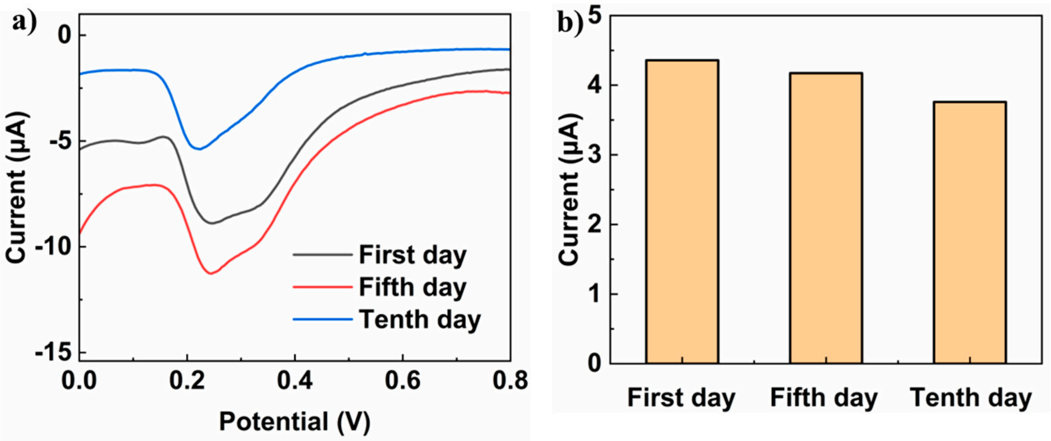

Figure S5. a) The CV curves of $Cu_3(HTTP)_2$/SPE in AA at different times; b) Bar chart of AA response current of $Cu_3(HTTP)_2$/SPE at different times.
